# The Role of Osteopathic Care in Gynaecology and Obstetrics: An Updated Systematic Review

**DOI:** 10.3390/healthcare10081566

**Published:** 2022-08-18

**Authors:** Nuria Ruffini, Giandomenico D’Alessandro, Annalisa Pimpinella, Matteo Galli, Tiziana Galeotti, Francesco Cerritelli, Marco Tramontano

**Affiliations:** 1 National Centre Germany, Foundation COME Collaboration, 10825 Berlin, Germany; 2 Centre pour l’Etude, la Recherche et la Diffusion Ostéopathiques “C.E.R.D.O.”, 00199 Rome, Italy; 3 Clinical-Based Human Research Department, Foundation COME Collaboration, 65121 Pescara, Italy; 4 Research Department, SOMA, Istituto Osteopatia Milano, 20126 Milan, Italy; 5 Fondazione Santa Lucia IRCCS, 00179 Roma, Italy

**Keywords:** osteopathic manipulative treatment, gynaecology, obstetrics, somatic dysfunction

## Abstract

Background: Many efforts are made to find safer and more feasible therapeutic strategies to improve gynaecological care. Non-pharmacological treatments, such as osteopathic interventions, could be used as complementary strategies to better manage different gynaecological conditions. This review aims to report the effectiveness of osteopathic treatment in the gynaecology and obstetrics field, updating the previous review published in 2016. The secondary aim was to elucidate the role of somatic dysfunction (SD) in osteopathic assessment and treatment procedures, as well as their health and economic implications. Methods: An electronic search was conducted in the following databases: Embase, MEDLINE (PubMed), and Science direct. All types of clinical studies published between May 2014 and December 2021 have been included: randomised controlled trial (RCT), controlled before/after, interrupted time series quasi RCT, case controls, case reports, case series, observational, clinical studies involving any type of osteopathic treatment, (standardised, semi-standardised or patients’ need-based treatment) performed alone or in combination with other treatments, were included). Results: A total of 76,750 were identified through database searching and other sources. After the removal of duplicates, 47,655 papers were screened based on title and abstract. A total of 131 full-text articles were consequently assessed for eligibility. Twenty-one new articles were included in the synthesis. A total of 2632 participants with a mean age of 28.9 ± 10.5 years were included in the review. Conclusions: Results showed an effectiveness of osteopathic care in gynaecology and obstetrics, but the studies were too heterogeneous to perform quantitative analysis and make clinical recommendations. Nevertheless, osteopathic care could be considered a safe complementary approach to traditional gynaecological care.

## 1. Introduction

Osteopathy is a form of manual medicine characterised by a whole-body intervention and the presence of a specific palpatory aspect to drive the treatment [1], the so-called somatic dysfunction [2]. The latter is defined as an “impaired or altered function of related components of the somatic (body framework) system: skeletal, arthrodial and myofascial structures, and their related vascular, lymphatic, and neural elements’’ [2,3]. Osteopathy is a wide-spread complementary medicine whose use has steadily increased over the past decade [4].

Several studies have demonstrated the efficacy of osteopathy in the treatment of musculoskeletal disorders [5] and different neurological diseases [6,7,8]. Although there is currently no consensus regarding the precise mechanisms of action underlying osteopathic therapies, new research indicates that Osteopathic Manipulative Treatment (OMT) can modify neurophysiological and cerebral activity [6,9,10,11,12]. Due to osteopathy’s emphasis on the individual, osteopaths and patients are interested in its application in the gynaecological and obstetrical fields [13].

Gynaecological disorders are very common globally, resulting in high direct and indirect costs [14]. To improve gynaecological care, continual efforts are made to identify safer, efficient and clinically relevant therapeutic strategies [15]. A systematic review on this topic has already been conducted in 2016 [16], but the authors argued that more robust research was needed to recommend osteopathic care for gynaecological and obstetric conditions. Reasons included the high heterogeneity of study design, the small number of included studies, and the high risk of bias in those studies. Ruffini and colleagues [16] emphasised the need for more studies with pragmatic methodologies, improved and more detailed descriptions of treatments performed, and systematic reporting of side effects.

Furthermore, no information regarding the osteopath’s assessment and treatment framework was provided in the previous review. How osteopaths manage the decision-making process and why they select the appropriate treatment are still under discussion [17].

Recently, a scoping review [18] focused on the role of somatic dysfunction (SD) in the osteopathic field and clarified some of these aspects by stating that treatments can be performed using different paradigms by considering a whole-body assessment and treatment (i.e., patients’ need-based treatment) or only one or some regions of the body in a standardised or semi-standardised protocol.

For these reasons, the primary aim of this review was to systematically report the effectiveness of osteopathic care in gynaecology and obstetrics, updating the previous review. The secondary aim was to elucidate the role of SD in osteopathic assessment and treatment procedures, as well as their health and economic implications.

## 2. Materials and Methods

This systematic review and meta-analysis was performed according to the PRISMA (Preferred Reporting Items for Systematic Reviews and Meta-Analyses) statement [19]. The protocol was regularly approved and published in an international prospective register of systematic reviews (PROSPERO, CRD42021253227). Compared to the protocol, the only change made in the present review is the exclusion of gray literature.

### 2.1. Search Strategy

The search was conducted on the following electronic databases from May 2014 to December 2021: EMBASE, PUBMED, and ScienceDirect. The search strategy was conducted using all combinations among an OMT term and an obstetric/gynaecological term. The following MeSH and free terms with the Boolean operator “AND” between the two terms were used: OMT category = cranial sacral treat*, craniosacral, osteopath*, spinal manipul*, manipul*, osteopath* treat*, mobiliz*, OMT, somatic dysfunction, visceral treatment, myofascial release, cranial field; obstetric/gynaecological category = dysmenorr*, female infertility, menopaus*, menstr*, childbirth, pelvic pain, pelvic floor, pregnan*, maternal-fetal, gravid*, labor, birth, climacteric, fertility, sterility*, fert*, hormon*, premenstrual, gynecol*, obstet*, perineum, puber* (puberty, puberal), incontinen* (incontinent, incontinence, post partum, ovulat* (ovulatory, ovulation), endometr* (endometrial, endometriosis), dyspareun*, PCOS, ovar* (ovary, ovarian).

### 2.2. Inclusion and Exclusion Criteria

All types of clinical studies (randomized controlled trial (RCT), multi RCT, controlled before/after, interrupted time series, quasi RCT, case controls, case reports, case series, observational), published between May 2014 and December 2021, were included. Clinical studies involving any type of OMT, standardised, semi-standardised or patients’ need-based treatment on any anatomical area, without regard for the type of techniques used, alone or in combination with other treatments, were included. Included languages were English, Italian, French, German, and Spanish. Types of control included sham therapy, both manual and through devices, time control and other treatments. Population inclusion criteria were: women with gynecologic and obstetric conditions, including but not limited to pregnancy, labor, infertility, dysmenorrhea, pelvic pain and menopause.

Excluded studies were reviews of the literature, study protocols, commentaries, personal contributions, unpublished works, and studies applying any other form of manual medicine to the study group.

### 2.3. Data Items

The primary outcome of the present review was to evaluate the effectiveness of OMT in women with gynaecological and obstetrics disorders compared to any control group. Secondary outcomes were to quantify side effects associated with the osteopathic intervention, the health-economics implication of osteopathic care, and to investigate the type of osteopathic treatment and the use of SD in research trials. Data items for the osteopathic evaluation and treatment were the criteria to identify the SD, the techniques used and type of paradigm to perform the treatment (patient’s need, semi-standardised or standardised).

### 2.4. Selection Process

Titles and abstracts were identified, collected in the database and screened for relevance. In case of any doubt (e.g., no abstract available or unclear details), studies were admitted to the second step of the screening process. In this second stage, full texts were read and evaluated for inclusion in the review. When full texts and/or additional information were unavailable, corresponding authors were contacted via email. Both the data screening and selection process were independently performed by three reviewers (NR, GDA, AP). The eventual discrepancies were solved through discussion with an external arbiter (MT).

### 2.5. Data Extraction

The same reviewers extracted data from the included studies following a predetermined data extraction form. The form was filled in for bibliographic information on the included studies, study aims and design, methods and setting of recruitment, inclusion/exclusion criteria, informed consent, conflict of interest and funding, type of intervention and control, number of participants, characteristics at the baseline, setting of intervention, type of outcome and time points for assessment, results, and adverse events. The osteopathic intervention and the manual sham therapy were analysed following the “whats, hows and how-not-tos” descriptive principle [20] and the equality assumption (EA) between interventions was assessed. Data were reported by considering the role and use of SD in the assessment, treatment and time frame of the osteopathic treatment. Study risk of bias assessment.

The Revised Cochrane risk-of-bias (RoB) tool for randomised trials [21] was used to rank the RCTs. The RoB tool analyses the following five domains: randomization process, deviations from the intended interventions (effect of adhering to intervention), missing outcome data, measurement of the outcome, and selection of the reported result. The risk of bias can be ranked as low, or high, for some concerns according to the algorithm shared by the Cochrane Library.

Non-RCT and observational studies were critically assessed using the Methodological Index for Non-randomized Studies (MINORS) score [22], with a global ideal score of 16 for non-comparative studies and 24 for comparative studies. The MINORS score was then calculated as a percentage of the maximum score, considering a score of 8 or less to be poor quality, 9 to 14 moderate quality, and 15 to 16 good quality for non-comparative studies. For comparative studies, the categories referred respectively to 14 or less, 15 to 22, and 23 and 24.

Case reports were assessed with the Joanna Briggs Institute Checklist for Case Reports [23].

### 2.6. Data Analysis

An intention-to-treat analysis was planned, including all trials randomised to their original groups, regardless of whether or not the participants dropped off the trial. Regarding treatment effect measurements, it was aimed to describe results and differences between groups or time points. For continuous data, the mean, standard deviation (SD), and 95% confidence interval (CI) were reported; for dichotomous data, the relative risk (RR) and 95% CI were provided. If osteopathic treatment was protective compared to the control group, the relative risk was less than 1. An estimated pooled weighted mean of relative risks (RRs) was computed. I2 statistics was used to quantify heterogeneity. When studies were methodologically, clinically, and statistically comparable (qualitative evaluation of studies), the Mantel–Haenszel random-effect method was applied to conduct a meta-analysis [24] using Rev Man. 5. If included studies lacked pertinent data, their authors were contacted to obtain them. Whenever possible, summary and descriptive statistics were presented in lieu of meta-analysis.

### 2.7. Differences with the Previous Review

The present review excluded grey literature and introduced a quality assessment of non-randomized studies. To obtain a uniform assessment of all the included studies and to facilitate the update of the systematic review, the old review’s trials were re-analyzed using the new extraction form, the updated RoB Tool, and the quality assessment tools.

## 3. Results

**Figure 1:**
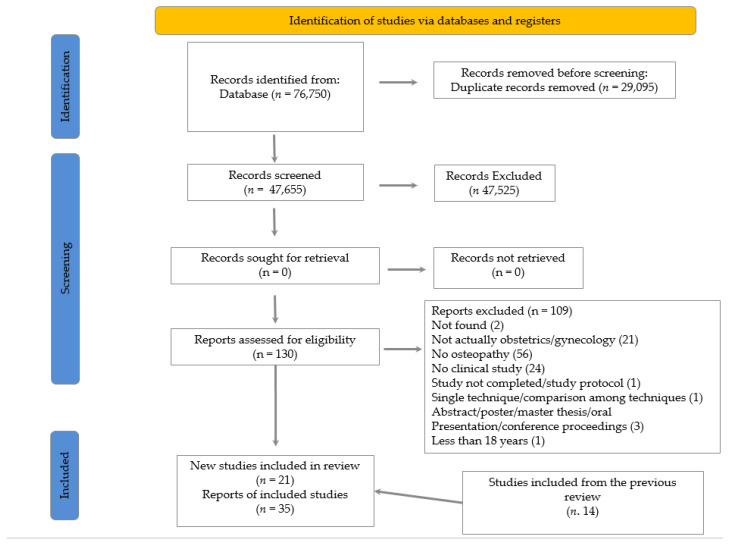
PRISMA 2020 flow diagram with the updated findings.

A total of 76,750 were identified through database searching. After duplicate removal, 47,655 papers were screened based on title and abstract. Then, 130 full-text articles were consequently assessed for eligibility, 109 articles were excluded for not respecting the inclusion criteria, or because full-texts were unavailable. In addition, 21 new studies were included in the qualitative synthesis (Figure 1). The type of study has been reported in Table 1. There were a total of 2632 participants with a mean age of 28.9 ± 10.5 years. The investigated clinical conditions have been reported in Table 2.

No new papers addressing the topic of infertility have been found in the literature with respect to the previous systematic review, while new articles were found about vulvodynia, endometriosis and PCOS.

### 3.1. Pregnancy

Three case reports [25,26,27] conducted in Europe and one RCT performed in the USA investigated the role of OMT in the pregnancy field. The RCT [28] known as the PROMOTE study lasted 5 years and included 400 women at the 30th gestational week, but 301 of them did not complete the foreseen protocol of visits. The results showed that OMT was superior in reducing pain and improving back function if compared to the usual care only, but no differences in back pain and function have been found between OMT and the other control condition (placebo ultrasound therapy). The OMT was considered safe since no side effects were reported.

Urbanek [27] investigated the effect of osteopathic treatment on a 32-year-old woman at the 23rd week of a high-risk twin pregnancy with polyhydramnios and on the verge of developing a feto-foetal transfusion syndrome. The relevant aspect of this case report was the immediate amelioration of objective parameters measured by the Doppler examination. After the first treatment (23rd week), the patient had no longer need to be hospitalised due to Doppler improvement. Six other OMTs were performed, and the Doppler examination constantly confirmed the normal development of monochorionic twins with a slight sign or without any sign of twin-to-twin syndrome. The twins were born after caesarean section at the 38th week with excellent Apgar scores. This case report showed that OMT could safely be used in high-risk twin pregnancies with polyhydramnios but further RCTs can be carried out to evaluate if it can ameliorate blood flow, growth and the well-being of both mother and children.

Schreiber [25] studied the effect of OMT on the 37-year-old primipara at the 28th week of pregnancy, exhibiting Low Back Pain (LBP) since the 26th week (VAS 4.5). The paper reported that the pain disappeared, but the second assessment of VAS was not implemented.

The case report of Russo and colleagues [26] evaluated the benefits of hypnotherapy and osteopathy in restoring a woman’s visual function after pregnancy. The 35-year-old patient reported a decrease in visual acuity after the two previous pregnancies. Visual acuity was measured through the Unidad Bueno–Matilla tool at baseline, after treatments, and at a 1-month follow-up. The double intervention contributed to the clinical improvement of visual acuity. The visual acuity rating passed from 93, 92 (respectively, right and left eyes) to 102, 101.

### 3.2. Labour

Two RCTs [29,30] and one pilot study [31] examined the potential benefits of osteopathy on labour-related outcomes. Both RCTs [29,30] were part of the previously reported PROMOTE study [28]. The objective of the first RCT [29] was to compare the incidence of high-risk status and labour and delivery outcomes in pregnant women who received an OMT protocol during the third trimester. Participants who received OMT were 2, 6 and 2, 3 times less likely to be classified as high risk than those in the UCO and PUT groups. Participants who received OMT were 2, 3 and 4 times more likely to experience prolonged labor than participants in the placebo ultrasound treatment and usual care only groups, respectively.

The outcome of the second RCT [30] was more strictly osteopathic. The study aimed to determine if compression of the fourth ventricle (CV4) could induce uterine contractions and labour as part of the OMT protocol used in the PROMOTE study. There were no statistically significant differences between the OMT group (in which all participants received CV4 technique) and the control groups concerning the development of a high-risk status or preterm birth.

In their pilot study, Martigano and colleagues [31] enrolled 100 women at the 34th week of pregnancy (mean age: 28 years) to determine whether labour management by osteopathic obstetricians could reduce overall labour duration, meconium-stained amniotic fluid, and the rate of caesarean delivery. Women managed by osteopathic obstetrics had a significantly shorter labour duration of 11.34 (range: 1.1–27.0) hours compared to 16.57 (range: 1.0–58.8) hours for women managed by allopathic obstetrics.

### 3.3. Postpartum

Two studies [32,33] were conducted on the topic of postpartum.

Schwerla and colleagues [32] examined the effectiveness of OMT in reducing postpartum LBP, pelvic girdle pain (PGP), and functional disability in women with a history of pregnancy-related LBP for at least three months after delivery. Participants were recruited from the general population. Women were assigned to an OMT group and a waitlist control group by means of external randomisation. Four sessions of osteopathic manipulative therapy were administered at 2-week intervals, with a 12-week follow-up. The OMT was individualised for each participant and based on the needs of the patients. The control group participants did not receive OMT during the 8-week study; instead, they were placed on a waiting list to receive OMT after the study. During the study period, they were not permitted to receive any additional treatment (i.e., medication, physical therapy, or other sources of pain relief). Pain intensity as measured by a visual analogue scale (VAS) and the impact of LBP on daily activities measured by the Oswestry Disability Index (ODI) were the primary outcome measures. VAS was found to be significantly enhanced in the OMT group; ODI was also significantly enhanced within the group but significantly lower at baseline in the OMT group. During the study period, no serious adverse effects were reported. On occasion, participants who received OMT reported feeling tired.

Kirk and colleagues [33] investigated the effect of visceral manipulation (VM) on diastasis recti abdominis (DRA). The ages of the patients were 33, 37, and 39 years old and all were positive for DRA based on inter-rectus distance (IRD) described as greater than 2 finger-width measurements at 1 out of 3 measurement sites: above, at, and below the umbilicus. Patients presented with low back pain, abdominal pain, vulvar burning and itching as their primary complaints, their symptoms appeared after the first pregnancy and worsened after the second one. Each patient received at least four VM treatments, which appear effective in reducing DRA levels in 3 women. IRD measurements improved to within two finger widths and remained stable for 6 to 16 months.

### 3.4. Pelvic Pain

One case report by Origo and colleagues [34] described the osteopathic management of a 40-year-old woman suffering from pudendal nerve entrapment after three proctological surgeries. The patient received 3 months of pharmacological treatment, followed by five patient need-based osteopathic sessions over 5 weeks. The outcome measures were collected before (T0) and after (T1) the pharmacological regimen, after OMT (T2) and at six months follow-up (T3). The results showed an important improvement after OMT in all outcomes considered: VAS (T0 = 10, T1 = 10, T2 = 1.8, T3 = 1.5), female National Institutes of Health Chronic Prostatitis Symptom Index (T0 = 34, T1 = 30, T2 = 7, T3 = 6), Oswestry Disability Index (T0 = 48, T1 = 29, T2 = 9, T3 = 5), and Tampa scale of kinesiophobia (T0 = 51, T1 = 41, T2 = 20, T3 = 17).

### 3.5. Vulvodynia

One case report by Giovanis and colleagues [35] described the effect of osteopathic treatment on a 40-year-old woman with vulvodynia and dyspareunia following a physical strain. The incident occurred a year before the treatments, and all other treatments, including physiotherapy, Botox injections, and antidepressants, had failed. Following a patient’s need-based treatment model, the osteopath administered 7 interventions over four months. Vulvar pain and depression were considered outcomes, and both were reported to have improved by the fourth month’s end.

### 3.6. Endometriosis

Five studies [36,37,38,39,40] examined the effect of osteopathic treatment on women diagnosed with endometriosis, including 1 prospective cohort study [36], 2 pilot studies [37,38], and 2 case reports [39,40].

In a prospective cohort study, Darai and colleagues [36] examined the effect of a standardised osteopathic treatment on 68 women (mean age 32 ± 6.2) with colorectal endometriosis. The French version of the SF-36 and a VAS for gynaecological, digestive, urinary, and general symptoms, such as radiating pain in the sacrolumbar region and asthenia, were utilised as outcomes. After treatment, 46 patients returned questionnaires, and the analysis revealed a significant improvement in quality of life and pain across all domains, excluding urinary symptoms. Subsequently, the authors conducted a subgroup analysis according to the severity of symptoms (cluster 1 had low severity, cluster 3 had high severity, and clusters 2 and 4 were described as “heterogeneous”), showing significantly improved symptoms in clusters 2 and 3.

Another prospective pilot study [37] investigated the effect of one osteopathic session on the quality of life of patients diagnosed with deep infiltrating endometriosis, as measured by the SF-36. Twenty-eight patients (ranging in age from 22 to 39) were included; twenty received OMT, and fifteen returned the questionnaire following the session. The outcomes indicate a substantial improvement in bodily pain, general health, vitality, social functioning, and mental health.

In their pilot study, Sillem et al. [38] performed semi-standardized OMT on a sample of 28 women (ages 20–65) with chronic pelvic pain unrelated to the menstrual cycle. Patients were assigned randomly to two groups (with and without endometriosis). The median number of sessions per patient was six (ranging from 1 to 24). Five women in the endometriosis group (EG) repeated the treatment, while one left the study. Five patients dropped out of the group without endometriosis (NEG), and 3 patients repeated the prescription. Ten women in the EG and 7 women in the NEG report an improvement in symptoms. Changes in symptoms were reported in response to “in-person or written satisfaction with treatment” questionnaires. Outcome measurement ambiguity and the number of sessions heterogeneity reduce the validity of the results.

One case report [39] described the osteopathic treatment of a 24-year-old woman with LBP and pelvic, low abdominal pain that recurred approximately every four weeks. Three months prior, the patient was diagnosed with endometriosis and began hormonal therapy. Following a patient’s need-based treatment model, the osteopath administered four OMT sessions, which resulted in an almost immediate reduction in symptoms. Interestingly, the hormonal therapy was discontinued after a doctor’s visit that revealed endometrial foci atrophy.

Goyal et al. [40] described the effects of OMT on a 29-year-old woman with menorrhagia, abdominal pain, leukorrhea, low back pain, and fatigue during her menstrual phase for at least three years. The ultrasound revealed a large uterus with minor endometriosis. The attempt to treat the symptoms with proton pump inhibitors, antidepressants, and anti-inflammatory drugs had failed. The patient received eight osteopathic treatment sessions over four weeks, but the intervention is not described in detail. The Endometriosis Health Profile-5 questionnaire (pre-OMT = 72/100, post-OMT = 26/100) and a VAS for menstrual pain (pre-OMT = 8.3/10, post-OMT = 3.9/10) were used to track the change in symptoms.

### 3.7. Dysmenorrhea

Two case reports [41,42] and one RCT [43] were conducted on women with dysmenorrhea.

Matsushita and colleagues [41] described the effects of osteopathic treatment on a 32-year-old woman with a sudden onset of grade 3 secondary dysmenorrhea, headaches, low back pain, mood changes, nausea, diffuse abdominal pain, constipation, and diarrhoea. The medical examinations revealed no cause for the dysmenorrhea, but because the patient was experiencing a stressful personal situation, psychosocial factors were considered as a possible cause for the symptoms. The patient received six OMT sessions based on the patient’s needs over five months and counselling on nutrition, exercise, yoga, and mindfulness meditation. The multidisciplinary approach reduced dysmenorrhea from grade 3 to grade 1, which was sustained at the follow-up interview one year after treatment.

Origo and colleagues [42] published a case report of a 37-year-old woman diagnosed with dysmenorrhea following laparoscopic cholecystectomy, who also presented pelvic pain and deep dyspareunia, both of which had worsened progressively over the previous 15 years. After four sessions of need-based OMT, the patient reported a 50% reduction in pain during the menstruation week (Global Pain Scale) and a 70% improvement in disability (Female Sexual Distress Scale, Working ability Location Intensity Days of pain Dysmenorrhea), and complete recovery from dyspareunia. Interestingly, when the gynaecologist performed a follow-up transvaginal ultrasound, the right ovary, which had previously been located behind the uterus, was discovered to be in the correct position.

One multicenter RCT [43] examined the effect of OMT on women diagnosed with primary dysmenorrhea (*n* = 29) versus a waiting list control group (*n* = 31). Over three months, the intervention group received two nonstandard osteopathic treatments per menstrual cycle. Compared to the control group, the OMT group demonstrated a statistically significant reduction in pain intensity (NRS), duration of pain, and the number of days with pain > 5 on the NRS, and a non-significant improvement in quality of life as measured by the SF-36. In the OMT group, NSAID consumption decreased by 75%, whereas it increased in the control group.

### 3.8. Polycystic Ovary Syndrome (PCOS)

Using the manipulation of Chapman points and viscerosomatic reflexes, a pilot study [44] describes the effects of OMT on sympathetic tone in women with PCOS. In the second phase of a larger study by the same group of authors, 25 women between the ages of 22 and 43 were recruited: 14 received semi-standardised OMT once per week, and 11 received osteopathic evaluation but no intervention. In both groups, the following outcomes were evaluated at baseline and 3 months: resting heart rate and blood pressure, heart rate recovery after 15 min of aerobic exercise, heart rate variability at rest, serum androgen levels, body mass index, fasting blood glucose and insulin levels, and menstrual cycle length. Nineteen women participated in the study. Comparing pre-intervention and post-intervention parameters, women with PCOS in the OMT intervention group demonstrated an improvement in post-exercise systolic blood pressure (135.8 ± 2.6 vs. 129.1 ± 2.5 mm Hg; *p* = 0.0002) and a trend toward heart rate recovery (23.2 ± 3.3 vs. 29.4 ± 2.2 s; *p* = 0.099). There were no significant improvements in the control group, or any other physiologic parameters evaluated. There were no significant improvements in the endocrine, metabolic, or reproductive parameters measured, although free testosterone was marginally lower after 3 months of weekly OMT (5.69 ± 1 vs. 4.64 ± 0.73 pg/mL).

### 3.9. Menopause

A clinical trial [45] evaluated the long-term effect of OMT on the symptoms and quality of life associated with menopause in women. Twenty patients were randomly divided into two groups and assigned to the needs-based treatment for the osteopathic patients’ group and the control group. Patients were given the MENopause-specific Quality of Life questionnaire (MENQol) and the Menopause Rating Scale (MRS). Only patients in the intervention group experienced a significant improvement in outcomes after three months of osteopathic intervention. Over more than nine months of follow-up, the treatment’s benefits were maintained. The results of the questionnaire administered before and after the follow-up do not differ significantly.

### 3.10. Somatic Dysfunction and Characteristics of the Treatment Session

**Figure 2:**
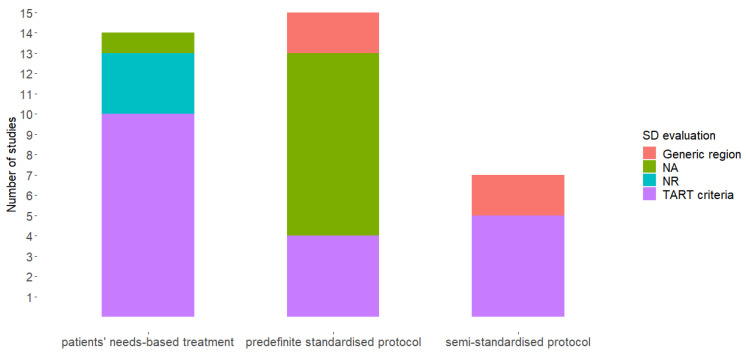
Somatic dysfunction detection methods. TART = Tissue texture, Asymmetry, Range of motion, Tenderness; NR = Not Reported; NA = Not Applicable; SD = Somatic Dysfunction.

We combined the results of the previous and current reviews and analysed the OMT characteristics of 35 studies. Twenty-two studies [25,29,31,32,33,34,35,38,39,40,41,42,43,44,46,47,48,49,50,51,52,53] accounted for SD. Tenderness, asymmetry, restricted motion, and tissue texture (TART criteria) were used to identify SD in 17 of the 22 studies [25,29,31,32,33,34,35,39,41,42,43,44,46,47,49,51,52]. In 4 of the 22 studies [38,40,42,53], the SD was considered a region rather than a specific structure (Figure 2).

**Figure 3:**
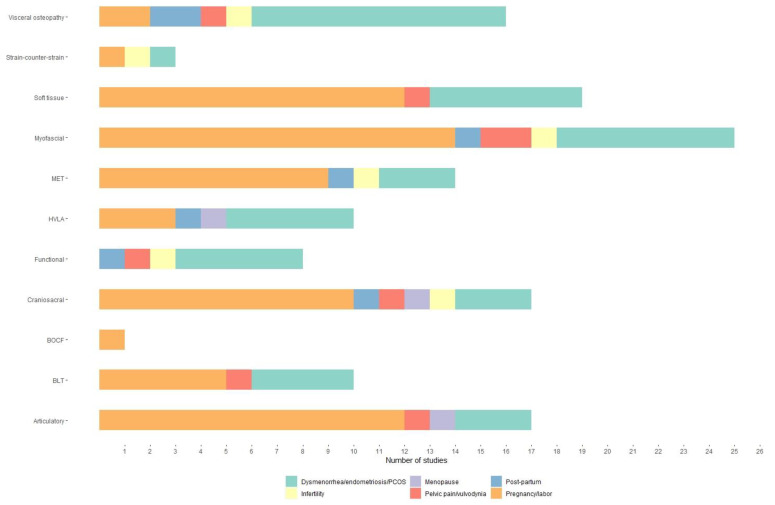
OMT Techniques used in the different clinical conditions. OMT = Osteopathic Manipulative Treatment; BOCF = Biodynamic model of Osteopathy in the Cranial Field; BLT = balanced ligamentous tension; MET = muscle-energy technique; HVLA = high-velocity low-amplitude.

In 14 out of 35 studies (40%) the treatment was based on the patient’s needs, in 7 (20%) studies on a semi-protocol and in 15 (43%) studies on a predefinite standardised protocol. Myofascial, soft tissue, cranial and articular were the most used techniques in the different conditions, as reported in Figure 3. These techniques were generally used in combination with each other. Indeed, myofascial techniques were often combined with muscle-energy, high-velocity low-amplitude (HVLA) articular, craniosacral and soft tissues techniques. In 1 study carried out on women with menopause symptoms [45], a semi-direct HVLA was used as an exclusive treatment [54]; as well, soft tissue has been chosen as a treatment for patients with labor-related symptoms [48].

Sixteen out of 35 studies reported the duration of the treatment session with a mean duration of 32 ± 15 min. Thirty studies reported the number of sessions received by patients (mean 5.8 ± 2.3).

### 3.11. Side Effects and Health Economics

We evaluated the results of the previous and current reviews. Five studies out of 35 reported information about the adverse effects of OMT. Hensel and colleagues [28] used the conversion to high-risk status as an indirect measure of safety, and they found no differences among the three groups. Indeed, the conversion to high-risk status occurred for 11 women of the usual care plus osteopathy group, 19 for placebo ultrasound treatment group, and 20 for usual care only group.

Schwerla and colleagues [32] reported no adverse events during the treatment period. Elden and colleagues [55] reported no serious adverse events, with 10 women in each group reporting disadvantages (temporarily increased PGP, elastic pelvic belt discomfort and drowsiness). In the remaining two studies, the OMT was well tolerated without complications [42,51].

Three studies [43,46,56] explored health economics without reporting its implications.

### 3.12. Quality Assessment

The Revised Cochrane tool on the risk of bias for randomised studies (RoB 2 2019) was used to classify the RCTs. The judgments formulated by the previous SR with the RoB 2011 were also reported (see Table 3). They were defined as low risk (Low), with some concerns or a high risk of bias (High). The quality assessment of some studies is updated using the RoB 2019 with respect to the previous review. Indeed, the study’s evaluation of Guthrie and colleagues [48] changed from High to Some concerns, Cleary [53] from Low/Moderate to Low, Licciardone and colleagues [47] from Moderate to Some concerns, and in another study from High to Low [46], and Hensel [57] from Unclear to High.

Non-RCT and observational studies were critically assessed using the Methodological Index score for non-randomized studies MINORS and quality were rated as low (Poor), medium (Moderate) and good (see Table 4). Due to the high clinical and methodological heterogeneity among the included studies, no meta-analyses were performed.

## 4. Discussion

This systematic review aimed to investigate the effectiveness of OMT in gynaecology and obstetrics, updating previous findings [16] with a focus on the efficacy, safety, and role of SD in osteopathic assessment and treatment. We could not perform a quantitative synthesis of the results due to the high level of heterogeneity, but we did describe the effectiveness of OMT for each clinical condition. Following the previous review [16], the results indicate an effectiveness of OMT on pregnancy-related pelvic pain and function compared to standard care, a mild protective effect on the development of high-risk status during the third trimester of pregnancy [29,57], but contradictory results regarding the reduction of CSD rate [37] and meconium-stained amniotic fluid [57]. A case series revealed that visceral manipulation effectively reduced postpartum diastasis recti abdominis with long-lasting results for up to sixteen months [33]. A pragmatic RCT [32] demonstrates significant improvement in PGP and functional disability after 4 sessions for women with postpartum LBP and pelvic pain. Two case reports described positive effects on pelvic pain and vulvodynia/dyspareunia, but due to the design of the study, the results remain anecdotal. Women with dysmenorrhea and endometriosis showed more consistent improvements, but the studies were too diverse to conduct a meta-analysis. Overall, only 2 studies [45,53] investigated the effect of OMT on menopause-related symptoms, but the low quality of the evidence precludes the development of recommendations. There were no new publications regarding infertility and subfertility.

From May 2014 to 2021, 21 new studies were released. It may seem to be a small number, but the previous review (from inception to April 2014) only included 14 published articles. In addition, the COVID-19 pandemic has made it challenging to conduct experimental studies. This demonstrates, in our opinion, a growing interest in gynaecological and obstetric conditions. Additionally, 9 of the 21 were case reports. On the one hand, case reports often are the methodology of choice to bring attention to a new field or a therapy that has not yet been explored. On the other hand, case reports are based on clinicians’ experiences embraced into the evidence-based medicine paradigm. These elements allow clinicians to understand the importance of sharing relevant clinical experiences with the scientific community to promote knowledge advances. However, we need to highlight that, although case reports are hypothesis-generating, they are still the first step of the pyramid of evidence, therefore showing low quality, low generalizability and minimal methodological aspects.

An intriguing result relates specifically to the specificity of osteopathy. Indeed, Hensel and colleagues [30] included the CV4 technique in the treatment protocol, which was previously deemed contraindicated during pregnancy because it was believed to induce uterine contractions. The contraindication originated from a small 1992 study [58] and rapidly became a “professional dogma” [28]. The sub-analysis [30] failed to demonstrate that CV4 induces preterm labour. This result paves the way for future research on this subject.

Despite the abundance of literature on the subject [20,59,60,61,62], the controls in the examined studies are frequently inadequately described, particularly for the manual placebo (or so-called “sham therapy”). When described, control procedures are not always set according to the literature-derived gold standard. This result is consistent with the findings of Cerritelli and colleagues [62], which found an extreme heterogeneity of controls and a general lack of description of controls in osteopathy trials. Future research should consider the debate surrounding the placebo/sham therapy concept in osteopathy and manual medicine in general [30]. Notably, 4 studies improved in terms of Bias Risk when evaluated with the RoB 2019 as opposed to the RoB 2011. Licciardone et al. [46] went from “high” to “some concerns”; Hensel et al. [57] went from “unclear” to “low”; Licciardone et al. [47] went from “moderate” to “some concerns”; and Cleary [53] went from “low/moderate” to “low”.

Compared to the previous review, the adverse effects have been more thoroughly described in more recent publications. However, it continues to be underreported. There was no improvement in the reporting of economic-related consequences. Safety and cost-effectiveness are essential factors for patients and physicians recommending any type of therapy within the healthcare system. Furthermore, in this review, we have reported information about the criteria to identify the SD, the different osteopathic techniques used in the gynaecological and obstetric conditions, and we analysed the different paradigms that are chosen for the assessment and treatment.

Indeed, according to a recent scoping review [18], the characteristics and use of SD and the type of osteopathic approach follow the same pattern as other clinical conditions. Taking into account the findings of the previous review, 14 out of 35 studies used a predetermined standard protocol, 14 used a patient-centred approach, and 7 used a semi-standard protocol. No correlation was identified between the type of treatment and the clinical conditions. This result highlights the heterogeneity of the study design and protocols in osteopathic research. Moreover, the application of SD varies significantly from study to study, confirming the need for an international consensus within the osteopathic practice community regarding a reliable assessment method that justifies the choice of treatment type and/or manual technique. The heterogeneity of validated systems for recording, collecting, and evaluating clinical palpatory findings is another important consideration. In fact, only 17 out of 25 studies considered TART criteria for SD detection.

### Strengths and Limitations

To the best of our knowledge, this is the first systematic review to report side effects, health economics, and the characteristics of the osteopathic treatments in all gynaecological and obstetric conditions. One limitation is that, despite the best effort, some studies may have been missed during the bibliographic search. Secondly, considering the heterogeneity of the included studies, no meta-analysis could be performed, and, therefore, clinical recommendations cannot be made. Furthermore, the broad review aims may have been better suited for a scoping review design.

## 5. Conclusions

The present systematic review shows an increase in the quantity and quality of published studies and a broader array of covered topics. Results showed an effectiveness of osteopathic care in gynaecology and obstetrics, but the studies were too heterogeneous to perform quantitative analysis and make clinical recommendations. Nevertheless, limited evidence suggests that osteopathic care is safe when complementary to traditional gynaecological care.

## Figures and Tables

**Table 1:** Number of study types included in the current review and in the previous one [16].

Type of Study	New Studies Included in Current Review (N.)	Studies Included in the Previous Review (N.)	Total of Studies (N.)
Case report	9	2	11
Case series	1	2	3
Pilot prospective observational study	2	\	2
Pilot study	1	\	1
Prospective cohort study	1	\	1
RCT	7	6	13
Controlled before–after study	\	1	1
Controlled prospective study	\	1	1
Observational Retrospective study	\	1	1
Retrospective case-control	\	1	1

RCT = Randomized Controlled Trial; \= no studies found; N = Number.

**Table 2:** Type of clinical condition and number of studies included in Ruffini et al. [16] study as compared to the current review.

Type of Clinical Condition	New Studies Included in This Review (N.)	Studies Included in the Previous Review (N.)	Total Studies (N.)
Pelvic pain	1	1	2
Vulvodynia	1	/	1
Dysmenorrhea	3	2	5
Endometriosis	5	/	5
PCOS	1	/	1
Infertility	/	2	2
Pregnancy	4	5	9
Labour	3	3	6
Post-partum	2	/	2
Menopause	1	1	2

PCOS = Polycystic Ovary Syndrome; / = no studies found; N = Number.

**Table 3:** Risk of Bias assessment in RCTs. The table shows the score from the previous review, which applied the old version of the RoB Cochrane Tool, and the score of the new one, which applied the most recent version. Old = studies included in the previous review; New = studies included in the actual review.

	Guthrie 1982 [48]	Cleary 1994 [58]	Licciardone 2010 [59]	Licciardone2013 [56]	Hensel 2013 [60]	Elden 2013 [53]	Molins-Cubero 2014 [61]	Schwerla 2014 [45]	Noccioli 2014 [47]	Hensel 2015 [30]	Schwerla 2015 [34]	Hensel 2016 [31]	Hensel 2019 [32]	Davis 2020 [46]
Review	Old	Old	Old	Old	Old	Old	Old	New	New	New	New	New	New	New
RoB, 2011	High	Low/ Moderate	Moderate	High	Unclear	Low	Low	NA	NA	NA	NA	NA	NA	NA
RoB, 2019	Some concerns	Low	Some concerns	Low	High	Low	Low	Low	Low	Low	Low	Low	High	Low

NA = Not applicable.

**Table 4:** Risk of Bias assessment for non-randomized and non-controlled studies based on MINORS, with a global ideal score of 16 for non-comparative studies and 24 for comparative studies. For non-comparative studies, the MINORS score was calculated as a percentage of the maximum score, with a score of 8 or less to be poor quality, 9 to 14 moderate quality, and 15 to 16 good quality. For comparative studies, the categories corresponded to 14 or less, 15 to 22, and 23 and 24.

	Score for Each Question												Tot.	Overall Judgement
	1	2	3	4	5	6	7	8	9	10	11	12		
King et al., 2003 [57]	2	1	0	2	0	2	2	0	1	2	1	2	15	Moderate
Grimaldi et al., 2008 [49]	2	2	2	2	0	0	0	0	NA	NA	NA	NA	8	Poor
Kramp et al., 2012 [55]	2	2	2	2	2	2	2	0	NA	NA	NA	NA	14	Moderate
Schorpp et al., 2013 [50]	2	1	2	1	0	2	2	0	NA	NA	NA	NA	10	Moderate
Kermorgant et al. 2013 [51]	2	1	2	2	2	2	2	0	NA	NA	NA	NA	13	Moderate
Noccioli et al., 2014 [47]	2	2	0	2	0	2	1	1	1	2	2	1	16	Moderate
Darai et al., 2015	2	2	2	1	0	0	0	0	NA	NA	NA	NA	7	Poor
Sillem et al., 2016	2	2	0	0	0	0	0	0	NA	NA	NA	NA	4	Poor
Darai et al., 2017 [38]	2	2	2	2	0	0	1	0	NA	NA	NA	NA	9	Moderate
Martingano et al., 2019 [33]	2	2	2	2	0	2	2	2	2	1	2	2	21	Moderate
Kirk et al., 2021 [35]	2	2	2	2	1	0	0	0	NA	NA	NA	NA	9	Moderate

NA = Not Applicable.

## Data Availability

Not applicable.
